# Supplementary material for: Investigating Unfavorable Factors That Impede MALDI-TOF-Based AI in Predicting Antibiotic Resistance
Source: Diagnostics (Basel). 2022 Feb 5;12(2):413. doi: 10.3390/diagnostics12020413 (PMC8871102; doi:10.3390/diagnostics12020413)
Supplement: Supplementary file 1 [file diagnostics-12-00413-s001.zip › diagnostics-1563280-supplementary.pdf]

Supplement

Table S1. Prediction performance of ML model during 2020/08-2020/10. Prediction performance was inferior between probability 0.4-0.48.

	Probability<0.40	0.4-0.48	>0.48
accuracy	0.9857	0.88	0.94

Figure S1. Illustration of grey zone definition in this study. Probability range between 0.40 to 0.48 was defined as grey zone.

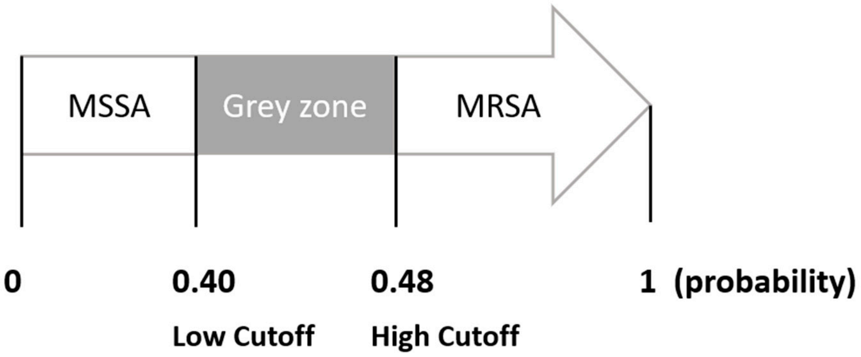

Table S2. Detailed peak numbers of the MALDI-TOF mass spectra in different groups.

	Minimum	First Quartile	Median	Third Quartile	Maximum
MRSA, correct	96	116	124.00	134.00	190
Grey zone	101	118.5	126	133.75	166
MSSA, correct	87	113	120	130	166
MRSA, incorrect	106	115	124	130	152
MSSA, incorrect	95	120	127.5	137.75	192

Table S3. Literature review of molecular typing of *S. aureus* in Taiwan.

Time	Research	Strain	Population	Sample size	Top 5 MLST		Other MLST
2012	Chen et al[1]	MSSA	- Community-onset infection<br>- 2 medical centers (Tri-Service General Hospital and Kaohsiung Medical University Hospital).<br>- a four-month	34	ST188<br>ST7<br>ST97<br>ST12<br>ST15	7 (20.6%)<br>6 (17.6%)<br>4 (11.8%)<br>4 (11.8%)<br>3 (8.8%)	10 (29.4%)

			collection period in 2006.				
2019	Chen et al[2]	MSSA	- Infected children (< 18y/o), excluding neonate<br>- Chang Gung Memorial Hospital, Linkou<br>- January 1, 2015 to December 31, 2015	59	ST15<br>ST188<br>ST7<br>ST508<br>ST5	6 (10.2%)<br>5 (8.5%)<br>4 (6.8%)<br>3 (5.1%)<br>2 (3.4%)	39(66.1%)
2014	Ho et al[3]	MSSA	- Blood culture of different patients<br>- China Medical University Hospital, Taiwan<br>- July 2008 to December 2009 for MSSA<br>- July to December 2008 for MRSA	14	ST15<br>ST59<br>ST188<br>ST12<br>ST6	4(28.6%)<br>3(21.4%)<br>3(21.4%)<br>2(14.3%)<br>2(14.3%)	-
		MRSA		22	ST5<br>ST239<br>ST59	10(45.4%)<br>7(31.8%)<br>5(22.7%)	-
2019	Wu et al[4]	MRSA	- Nasal swabs from patients >18y/o visiting the emergency department and health care workers in the ED and intensive care unit (ICU)<br>- 2 regional hospitals in Changhua<br>- February to June 2015	36	ST59<br>ST45<br>ST239<br>ST508<br>ST5	16(44.4%)<br>11(30.5%)<br>3(8.3%)<br>3(8.3%)<br>1(2.7%)	2 (5.5%)
2019	Peng et al[5]	MRSA	- MRSA isolates from cellulitis and from osteomyelitis	221	ST59<br>ST8<br>ST239<br>ST45	91(41.2%)<br>46(20.8%)<br>35(15.8%)<br>13(5.9%)	25(11.3%)

			patients.<br>- Chang Gung Memorial Hospital, Chiayi<br>- 2016 to 2018		ST30	11(5.0%)	
2012	Wang et al[6]	MRSA	- blood culture of different patients<br>- 9 medical centers, including in northern (hospitals N1, N2, N3), central (hospitals C1, C2, C3), and southern (hospitals S1, S3) and one in eastern (hospital E1) part of Taiwan.<br>- March to August, 2003	157	ST239<br>ST59<br>ST241<br>ST5<br>ST573	99(63.0%)<br>27(17.2%)<br>12(7.6%)<br>9(5.7%)<br>4(2.5%)	6 (3.8%)

References

1. Chen FJ, Siu LK, Lin JC, Wang CH and Lu PL. Molecular typing and characterization of nasal carriage and community-onset infection methicillin-susceptible Staphylococcus aureus isolates in two Taiwan medical centers. *BMC Infect Dis*. 2012; 12: 343.
2. Chen YJ, Chen PA, Chen CJ and Huang YC. Molecular characteristics and clinical features of pediatric methicillin-susceptible Staphylococcus aureus infection in a medical center in northern Taiwan. *BMC Infect Dis*. 2019; 19: 402.
3. Ho CM, Lin CY, Ho MW, Lin HC, Peng CT and Lu JJ. Concomitant genotyping revealed diverse spreading between methicillin-resistant Staphylococcus aureus and methicillin-susceptible Staphylococcus aureus in central Taiwan. *J Microbiol Immunol Infect*. 2016; 49: 363-70.
4. Wu TH, Lee CY, Yang HJ, et al. Prevalence and molecular characteristics of methicillin-resistant Staphylococcus aureus among nasal carriage strains isolated from emergency department patients and healthcare workers in central Taiwan. *J Microbiol Immunol Infect*. 2019; 52: 248-54.
5. Peng KT, Huang TY, Chiang YC, et al. Comparison of Methicillin-Resistant Staphylococcus aureus Isolates from Cellulitis and from Osteomyelitis in a Taiwan Hospital, 2016-2018. *J Clin Med*. 2019; 8.
6. Wang WY, Chiueh TS, Sun JR, Tsao SM and Lu JJ. Molecular typing and phenotype characterization of methicillin-resistant Staphylococcus aureus isolates from blood in Taiwan. *PLoS One*. 2012; 7: e30394.
